# Mechanisms of circular RNA degradation

**DOI:** 10.1038/s42003-022-04262-3

**Published:** 2022-12-09

**Authors:** Longxin Ren, Qingshan Jiang, Liyi Mo, Lijie Tan, Qifei Dong, Lijuan Meng, Nanyang Yang, Guoqing Li

**Affiliations:** 1 The Hengyang Key Laboratory of Cellular Stress Biology, Institute of Cytology and Genetics, Hengyang Medical School, University of South China, Hengyang, 421001 Hunan China; 2 Department of Otolaryngology, The First Affiliated Hospital, Hengyang Medical School, University of South China, Hengyang, Hunan 421001 China; 3 Department of Ultrasonography, Second Affiliated Hospital, University of South China, Hengyang Hunan, 421001 China

**Keywords:** RNA splicing, Non-coding RNAs, RNA, RNA decay

## Abstract

Circular RNAs (CircRNAs) are a class of noncoding RNAs formed by backsplicing during cotranscriptional and posttranscriptional processes, and they widely exist in various organisms. CircRNAs have multiple biological functions and are associated with the occurrence and development of many diseases. While the biogenesis and biological function of circRNAs have been extensively studied, there are few studies on circRNA degradation and only a few pathways for specific circRNA degradation have been identified. Here we outline basic information about circRNAs, summarize the research on the circRNA degradation mechanisms and discusses where this field might head, hoping to provide some inspiration and guidance for scholars who aim to study the degradation of circRNAs.

## Abstract (web summary)

In this Perspective, Longxin Ren and co-authors summarise the role of circular RNAs in disease and discuss recent insights into how circular RNAs are metabolised in the cell.

## Introduction

CircRNAs have become a novel research focus in recent years. However, the first circRNA molecules, viroids, were discovered as early as 1976^1^. Later, the widespread existence of circRNAs was discovered in highly diverged eukaryotes with the development of RNA sequencing and bioinformatic tools^2^. CircRNAs are a class of noncoding RNAs produced by a unique spliceosomal machinery. Researchers have studied the biogenesis and function of circRNAs^3,4^ and have achieved considerable success. An increasing number of studies have confirmed the effect of circRNAs on various diseases, especially cancer^5^. However, the path of research into the degradation of circRNAs is still being explored.

In this Perspective, we first briefly introduce circRNAs, mainly the biogenesis of circRNAs and the effect of circRNAs in various diseases. Next, we classify the identified degradation pathways of circRNAs and discuss the relationship between circRNAs and the substances involved in circRNA degradation. Finally, we summarize and point out the possible problems in the study of circRNA degradation and discuss possible future developments in this field.

## Main text

### Overview of circRNAs

#### Biogenesis of circRNAs

CircRNA biogenesis relies on the canonical spliceosomal machinery and is cell-type specific^6^. It is well established that ordinary linear splicing connects an upstream 5’ splicing site to a downstream 3′ splicing site, while the main assumption of backsplicing is that intron sequences are looped by joining the downstream 5′ splicing site to the upstream 3′ splicing site. Some *cis*-acting elements (such as Alu elements), *trans*-acting splicing factors, and RNA-binding proteins contribute to the progression of backsplicing (Table 1). In addition, lariats that form during exon skipping can contribute to circRNA biogenesis^4^. A majority of circRNAs are formed by backsplicing of exons, while a small part is formed by introns and intergenic regions^7^. Li Lie et al. also provided a well-defined model system to understand exon definition and backsplicing without needing a different spliceosome for each process^8^.

**Table 1:** Factors influencing circRNA biogenesis.

Factors	Effect	References
Alu elements	Inverted repeated Alu elements form dsRNA stem-loop structures to promote circRNA formation.	^66^
Quaking (QKI)	QKI binds upstream and downstream of the circRNA-forming exons to promote circRNA formation.	^67^
RNA-binding protein fused in sarcoma (FUS)	FUS binds to the introns flanking the backsplicing junctions to regulate circRNA biogenesis, and FUS can act both as an activator and a repressor of splicing.	^68,69^
Adenosine deaminase acting on RNA (ADAR)	ADAR reduces the formation of double-stranded structures in pre-mRNA, thereby reducing the stability of Alu elements and circRNA biogenesis.	^66,70^
DExD/H-box helicase 9 (DHX9)	DHX9 reduces circRNA formation by interacting with ADAR and recognizing RNA double-stranded structure.	^71^
Nuclear factor 90 and its 110 isoforms (NF90 and NF110)	NF90 and NF110 promote circRNA biogenesis by associating with intronic RNA pairs juxtaposing the circRNA-forming exons.	^72^
Heterogeneous nuclear ribonucleoproteins (HnRNPs) and serine–arginine (SR) proteins	HnRNPs and SR proteins work in concert to regulate circRNA biogenesis.	^64^
Nudix Hydrolase 21 (NUDT21)	NUDT21 regulates RNA cyclization through the interaction with the UGUA motif.	^73^
Neurotumor ventral antigen 2 (NOVA2)	NOVA2 binds to flanking introns of circRNAs loci to promote circRNAs biogenesis.	^74^

#### The effects of circRNAs on diseases

Current studies have revealed the functions of circRNAs, which include acting as miRNA sponges, acting as protein sponges, enhancing protein function, acting as scaffolds to mediate complex formation between specific enzymes and substrates, and recruiting proteins to specific locations^4^. Furthermore, as noncoding RNAs, some circRNAs have the ability to encode proteins^9^, such as circMAP3K4^10^.

Because of these numerous functions, circRNAs also have a crucial role in various diseases. For example, circHIPK3 plays an important role in angiogenesis and various types of cancers. circHIPK3 is closely associated with cell proliferation, migration, invasion, and autophagy^11,12^. The upregulation of circHIPK3 expression induced by tumor necrosis factor (TNF) can lead to a significant upregulation of cell migration ability and the rate of capillary-like structure formation, indicating that circHIPK3 has a proangiogenic effect on angiogenesis in the inflammatory microenvironment^13^. The upregulation of circHIPK3 expression also promotes the repair and renewal of intestinal epithelial cells after injury, while silencing of circHIPK3 inhibits the recovery of epithelial cells and the growth of intestinal organoids^14^. In lung cancer, the overexpression of circHIPK3 sponges miR-124 regulates the level of downstream target proteins and promotes lung cancer cell survival and proliferation^15^. Moreover, circHIPK3 is highly expressed in nasopharyngeal carcinoma tissues, and the reduction of circHIPK3 significantly inhibits tumor growth and metastasis in vivo, and further studies have revealed that circHIPK3 can act as a molecular sponge to bind miR-4288, preventing ETS transcription factor 3 silencing by miR-4288 and promoting nasopharyngeal carcinoma progression^16^. In hepatocellular carcinoma (HCC), circHIPK3 acts as a miR-124 sponge and regulates the expression of the miR-124 target gene aquaporin 3 (AQP3). AQP3 is upregulated in HCC tissues and negatively correlated with miR-124 expression. Overexpression of miR-124 decreased the expression of AQP3. Silencing circHIPK3 inhibited hepatocellular carcinoma cell proliferation and migration by downregulating the expression of AQP3^17^.

Many other circRNAs also play a role in diseases. CircSLC7A11 significantly accelerated the progression and metastasis of HCC through the circSLC7A11/miR-330-3p/CDK1 axis^18^. In diabetic kidney disease, circACTR2 may be a potential therapeutic target to inhibit regulated cell death and subsequent fibrotic remodeling^19^. Circular RNA-ZNF532 influences retinal pericyte coverage and vascular permeability through competitive binding with miR-29a-3p, which is associated with diabetic retinopathy^20^. Downregulation of circTLK1 inhibits cell proliferation, metastasis, and promotes apoptosis in renal cell carcinoma through miR-495-3p/CBL modulation^21^. In cardiovascular disease, circYAP reduces actin aggregation efficiency and inhibits cardiac hypertrophy^22^, and circFndc3b interacts with RNA-binding protein FUS to reduce myocardial cell apoptosis and improve myocardial function^23^. Moreover, knockdown of circTLK1 and overexpression of miR-335-3p can improve symptoms of neurological defects caused by acute ischemic stroke and protect neurons from damage^24^. CircARID1A can regulate autism spectrum disorder risk genes by sponging miR-204-3p^25^. A recent study has investigated the use of circRNAs in vaccine production because circRNA vaccines have the advantages of good thermal stability, high expression of coded antigens, and wide applicability. A corresponding circRNA vaccine has been successfully designed to resist the infection of novel coronavirus and its mutant strains^26^.

As mentioned above, the development of many diseases is associated with aberrant expression of circRNAs. The specific reason for this process is still unclear, but it is certain that the aberrant occurrence of key components in the biogenesis and degradation of circRNAs will disrupt the balance of circRNA expression. For example, in HCC, the expression of DHX9 is significantly increased, which leads to the decrease of circRNA cSMARCA5 expression and inhibits the proliferation and migration of HCC cells^27^. Systemic lupus erythematosus (SLE) patients showed reduced circRNA expression accompanied by spontaneous RNase L activation, and further studies revealed that circRNA was degraded by activated RNase L^28^. However, the general process of circRNA degradation is still elusive. At present, only some circRNA degradation pathways have been found, and the link between circRNA degradation and related diseases remains to be explored.

### The degradation of circRNAs

#### Correlation between endonuclease activity and circRNA degradation

**Fig. 1:**
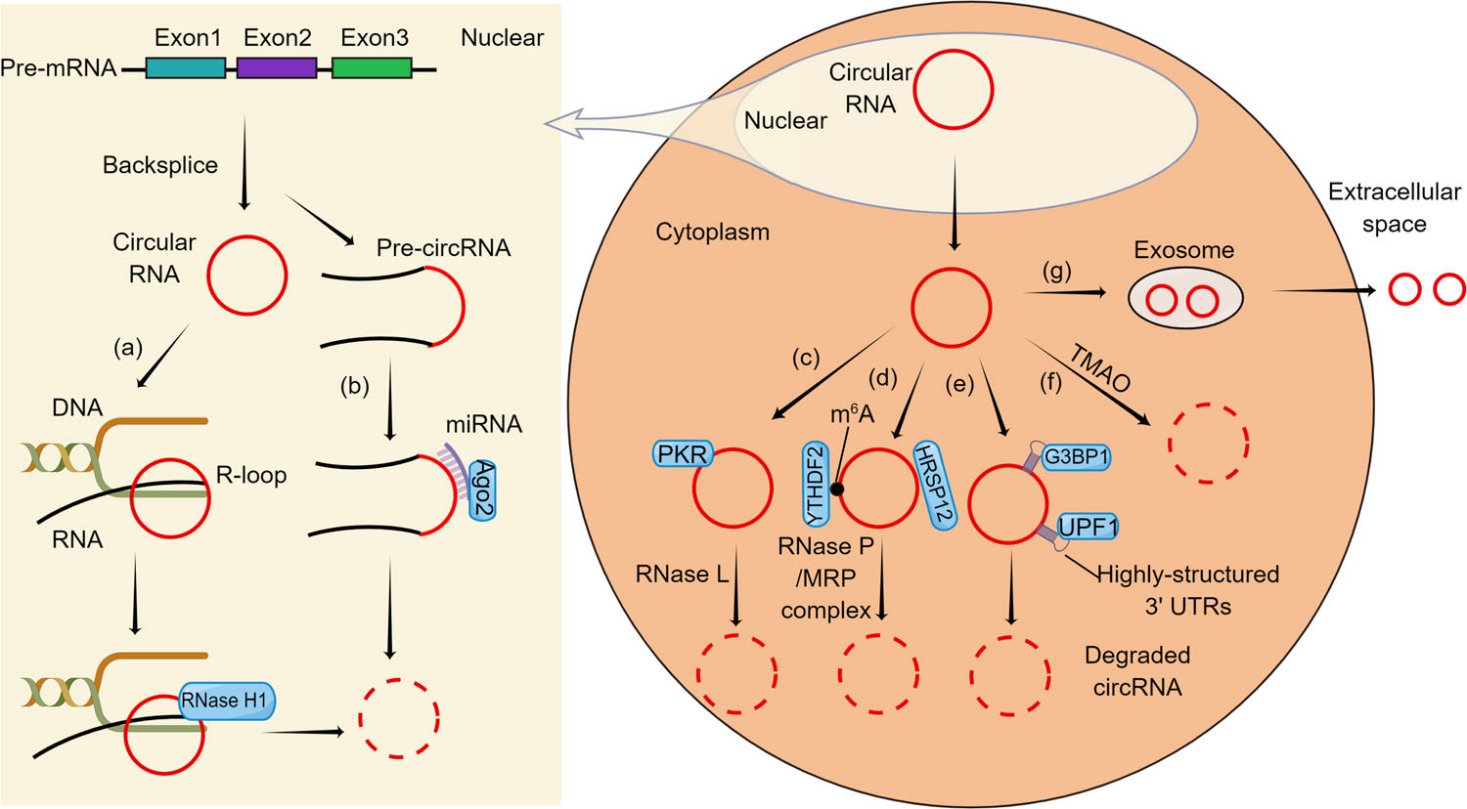
A model that shows several pathways of circRNAs degradation by Figdraw (www.figdraw.com). **a** RNase H1-mediated circRNA degradation; **b** Ago2-mediated circRNA degradation; **c** RNase L-mediated circRNA degradation; **d** m^6^A-mediated circRNA degradation; **e** structure-mediated circRNA degradation; **f** TMAO-mediated circRNA degradation; **g** exosome-mediated circRNA degradation.

First, some endonucleases participate in circRNA degradation. Some circRNAs can form R-loops with DNA at their expression sites, such as *ci-ankrd52*, which maintains a locally open secondary structure and forms a stable R-loop with template DNA. However, this kind of R-loop is subject to RNase H1 cleavage, and *ci-ankrd52* can be degraded via RNase H1 (Fig. 1a)^29^. RNase L is widely believed to restrict viral protein synthesis by cleaving host rRNA and viral mRNA, leading to translation block and viral mRNA degradation^30^. At the same time, circPTPN22 was shown to correlate with miRNAs and mRNAs related to immune regulation, including SLE development^31^. On this basis, it was revealed that endogenous circRNAs tend to form 16–26 bp imperfect RNA duplexes and act as inhibitors of double-stranded RNA (dsRNA)-dependent protein kinase (PKR) associated with innate immunity^28^. Moreover, PKR plays a key role in the viral infection response and intracellular homeostasis by regulating the translation of mRNAs. After dsRNA binds to PKR, PKR is activated through homozygous dimerization and autophosphorylation of Thr446 and Thr451 residues^32^. Upon inflammation or virus infection, circRNAs are globally degraded by activated RNase L, releasing PKR for its aberrant activation, as seen in SLE patients with reduced degradation of circRNAs leading to increased aberrant activation of PKR (Fig. 1c)^33^.

#### Correlation between Ago2 and circRNA degradation

Ago2 is also involved in the degradation of certain circRNAs. Ago2 is a member of the Argonaute family, which is highly conserved between species and widely expressed in different tissues^34^. In 2011, researchers found that miR-671 clears CDR1as in an Ago2-dependent manner^35^. In recent years, miRNA-1224 was shown to splice circRNA-Filip1l in an Ago2-dependent manner. Specifically, miRNA-1224 is an upstream regulator of the circRNA-Filip1l factor, and Ago2 recognizes and cleaves the complex formed by miRNA-1224 and pre-circRNA-Filip1l, resulting in a reduction in mature circRNA-Filip1l in the spinal nucleus^36^. In contrast, miRNA-671 differs from miRNA-1224 because miRNA-671 binds almost completely to circRNA-Cdr1as and forms the miRNA-671-circRNA-CDR1as complex in the nucleus, and then Ago2 cleaves and degrades circRNA-CDR1as after recognizing the complex (Fig. 1b)^37^.

#### Correlation between GW182 and circRNA degradation

In addition to Ago2, GW182 is also involved in the degradation of circRNAs. Depletion of GW182 can lead to the accumulation of steady-state circdati and circlaccase2 transcripts, while depletion of GW182 does not affect most nascent circRNAs. GW182 proteins consist of an Ago-binding domain (ABD), a ubiquitin-associated domain (UBA), a glutamine-rich domain (Q-rich), a middle region (Mid), an RNA-recognition motif (RRM), and a C-terminal region (C-term). It is believed that the ABD of GW182 mediates the interaction with Ago in the RNAi pathway, while ABD, UBA, and a Q-rich protein play an important role in the localization of GW182 to the P-bodies, suggesting that GW182 may regulate the degradation of some circRNAs in an Ago-slicer- or P-body--independent manner and the mid domain of GW182 also contributes to circRNA degradation^38^.

#### Correlation between m^6^A modification and circRNA degradation

N6-methyladenosine (m^6^A) is an internal modification associated with eukaryotic mRNAs and ncRNAs, and m^6^A-modified circRNA-SORE maintains sorafenib resistance in hepatocellular carcinoma by regulating the *β*-catenin signaling pathway^39^. Further, the presence of m^6^A modification was identified on circ3823 and the m^6^A modification was shown to be involved in regulating the degradation of circ3823^40^. To clarify the degradation process of circRNAs by m^6^A, researchers described endoribonucleolytic cleavage of m^6^A-containing RNAs via a YTHDF2-HRSP12-RNase-P/MRP pathway. YTHDF2 is a YTH-domain-containing protein that can recognize and destabilize m^6^A-containing RNAs. HRSP12 is a human heat-responsive protein 12. Eukaryotic RNase-P and its close relative RNase MRP are essential ribonucleoprotein complexes that function as endoribonucleases. The m6A-containing circRNAs associate with YTHDF2 in an HRSP12-dependent manner. HRSP12 functions as an adapter to bridge YTHDF2 and RNase-P/MRP, eliciting rapid degradation of YTHDF2-bound circRNAs (Fig. 1d)^41,42^.

#### Structure-mediated circRNA degradation by UPF1 and G3BP1

Structure-mediated RNA decay (SRD) is a unique pathway that regulates highly structured RNA (RNA with a 3′ untranslated region forming a highly folded structure in vivo) via two RNA-binding proteins, up-frameshift protein 1 (UPF1) and Ras-Gap-SH3 domain-binding protein 1 (G3BP1). Highly structured circRNAs are selectively upregulated upon depletion of G3BP1 or UPF1 in multiple cell lines, indicating that highly structured circRNAs may be regulated by the SRD mechanism. Further experiments revealed that UPF1 and G3BP1 could bind to highly structured base-pair regions of circRNAs and direct circRNA degradation. This suggests that there may be a circRNA degradation pathway similar to SRD in mRNA but slightly different from that in mRNA. The regulation of these highly structured circRNAs requires the RNA-binding and helicase activity of UPF1, as well as the RNA-binding and S149 phosphorylation site of G3BP1 (Fig. 1e)^43^. Moreover, the ability of UPF1 and G3BP1 to bind highly structured and poorly structured circRNAs is slightly different, and the specific reasons are not clear.

#### Correlation between TMAO and circRNA degradation

The gut microbiota (GM) can transform various dietary nutrients into trimethylamine (TMA). Most of the TMA enters the circulatory system and is subsequently oxidized to trimethylamine-n-oxide (TMAO) by hepatic flavin-containing monooxygenase (FMO)^44^. Furthermore, a high-sugar and high-fat (HSHF) diet could induce GM in mice, increase the TMAO level, and lead to changes in circRNA expression profiles. Further studies have conclusively shown that some circRNAs might be sensitive signaling molecules responsive to TMAO. These data have suggested that TMAO might influence the formation and degradation of host circRNAs (Fig. 1f)^45^.

#### Correlation between exosomes and circRNA degradation

In addition to being degraded within the cell, circRNAs can also be excreted from the cell. One study found large amounts of intact and stable circRNAs in human serum exosomes^46^, and exosomal circRNAs have also been suggested as a novel diagnostic biomarker in the early stages of cancer and a therapeutic target in further cancer treatment^47^. Further studies have revealed that circRNAs can be eliminated from cells by extracellular vesicles (EVs), such as exosomes or microvesicles, and the key evidence for this conclusion is that circRNAs are easily detected in EV preparations, and known circRNAs can be recovered from EVs. CircRNAs are also more abundant in EV preparations than their linear counterpart molecules, and this finding supports the idea that the release of circRNAs from cells into the extracellular space by EVs may be a mechanism by which cells eliminate circRNAs (Fig. 1g)^48^.

#### Potential association with circRNA degradation

An interesting question is a potential relationship between circRNA degradation and the nuclear export of circRNAs. CircRNAs are produced in the nucleus, while most circRNAs are distributed in the cytoplasm. How are they transported into the cytoplasm? It has been found that the nuclear export mode is determined by the lengths of mature circRNAs. Specifically, human URH49 regulates the nuclear export of short circRNAs, and *Drosophila* Hel25E and human UAP56 regulate the nuclear export of long circRNAs^49^. m6A modification also affects the nuclear export of circRNAs^50^; for example, YTHDC1 promotes the nuclear export of m6A-modified circNSUN2^51^. As mentioned above, m6A modification is also involved in the degradation of circRNAs, but no clear link has been found between the length of circRNA and degradation. Further understanding of the length measurement mechanism of circRNAs may help to discover some unknown links^52^. In recent years, it has also been found that EIF4A3 promotes the nuclear output of circPRKCI in triple-negative breast cancer cells^53^, and SRSF1(an SR protein) inhibits the output of circRNA^54^. Exportin 4 (XPO4) plays a role in the output of exonic circRNAs (ecircRNAs), and the deficiency of XPO4 leads to the nuclear accumulation of ecircRNAs, which then form harmful R-loops^55^. As mentioned earlier, circRNAs with high GC content will also form R-loops, and cells can recruit RNase H1 to degrade circRNAs in such R-loops^29^. Are there any structural similarities between the two types of R-loops, and are there any similar degradation mechanisms? This may provide a new way to study the relationship between ecircRNAs nuclear export and degradation.

Furthermore, a negative correlation between global circRNA abundance and cell proliferation has been investigated, and it is reported that a global reduction in circRNA abundance in colorectal cancer cell lines and cancer compared to normal tissues. The correlation of global circRNA abundance and cell proliferation was validated in a noncancerous proliferative disease, idiopathic pulmonary fibrosis, and ovarian cancer cells compared to cultured normal ovarian epithelial cells and 13 normal human tissues. This negative correlation appears to be a general principle in human tissues. These authors also made a simple hypothesis about how circRNAs accumulate in nonproliferating cells, namely, that circular and linear RNAs are synthesized by a gene dependent on a certain ratio of specific splicing events. CircRNAs display stability that far exceeds that of linear RNA^56,57^. During proliferation, both linear RNA and circRNA are uniformly distributed into daughter cells while being expressed, allowing a constant ratio of the two, with no accumulation of circRNAs in individual cells. In contrast, in nonproliferating cells, linear RNA is continuously produced and degraded, maintaining a constant expression level, but circRNA continues to accumulate in individual cells due to its stability^58^. Whether this relationship between circRNA abundance and cell proliferation is related to circRNA degradation is unclear.

#### Methods applied in research on circRNA degradation

To explore circRNA degradation, RNA-seq is a very routine method, which can help us identify and analyze the expression levels of circRNAs in organisms^31^. The microarray can also be used to identify circRNAs, which is more efficient than RNA-seq, and it can analyze reverse splice sites of circRNAs, but only known circRNAs can be detected^59^. NanoString nCounter is a multiplex nucleic acid hybridization technique that can be used in basic circRNA studies and clinical circRNA signaling studies^60,61^. Based on Illumina sequencing, some researchers have developed template-dependent multiplex displacement amplification, which can help discover circRNAs expressed at low levels and can be applied to any unannotated genomic organism^62^. Once the sequencing results are available, the relevant data can be first looked up in a database, and various circRNA databases (CircFunBase, CircR2Disease, etc.) are currently being created to query the expression levels of the target circRNAs in various cells and the associated signaling pathways. Then, the quantitative data of circRNA expression can be measured by RT-qPCR for validation. Northern blotting is also a standard technique for the validation of circRNAs^63^. In addition, many RNA interferences (RNAi)-based methods are used to interfere with circRNA expression, and target circRNAs can also be overexpressed by constructing circRNA overexpression vectors^14,64^. In situ hybridization (ISH) is also common in circRNA studies and can be used to visualize circRNA in cells^15^. If we consider studying circRNA-protein interactions, RNA pull-down, RNA-binding protein immunoprecipitation (RIP), and chromatin immunoprecipitation (ChIP) are currently popular experimental methods^16,23,40^. Moreover, the CRISPR-Cas9 system can be used to generate circRNA knockout (KO) mouse models, which can be used to study circRNA degradation at the animal level^37^. The experimental methods for circRNA degradation are still increasing with each passing day.

## Outlook

As discussed above, circRNAs play an important role in many diseases, such as congenital immune diseases, and various cancers. Furthermore, circRNA vaccines have been effective in the novel coronavirus pandemic currently sweeping the world. CircRNAs play an important role in the biomedical field as biomarkers with the potential to treat cancer and other diseases. Through RNA sequencing, we can easily identify a large number of circRNAs in different tissues and diseases, we still know little about the metabolic mechanisms of circRNAs. Although we have mentioned several pathways of circRNA degradation (Fig. 1 and Table 2), questions remain. For example, R-loop-coupled circRNA degradation and R-loop resolution mechanisms may not play a dominant role when circRNAs accumulate abnormally and pathologically^29^. Additionally, it is noteworthy that many circRNAs do not contain potential microRNA target sites for inducing Ago2 cleavage^47^, and few circRNAs exhibit the expected miRNA sponge properties^65^. Furthermore, although multiple pathways and hypotheses have been presented to regulate circRNA degradation, the details of these pathways are still unclear. For instance, future research may elucidate whether there are special enzymes or pathways for circRNA degradation and whether there is a single pathway for all circRNA degradation. Finally, we believe that with the continuous development of new experimental techniques and the unremitting exploration of researchers, the mechanisms of circRNA degradation will be thoroughly studied sooner or later. It is hoped that in future studies, these degradation mechanisms can be applied in related disease research to promote the degradation of circRNAs capable of producing diseases and inhibit the degradation of circRNAs capable of inhibiting diseases, as a means to evaluate the clinical role of these mechanisms.

**Table 2:** Pathways of circRNAs degradation.

Degradation pathways	Location	Interacting molecule	Related disease	Reference number
Endonuclease-mediated degradation	Nuclear/cytoplasm	RNase H1, Template DNA strand / RNase L, PKR	Systemic lupus erythematosus	^29,33^
Ago2-mediated degradation	Nuclear	miRNA, Ago2	Chronic inflammatory pain	^35–37^
GW182-mediated degradation	\	GW182	\	^38^
m^6^A modification-mediated degradation	Cytoplasm	YTHDF2, HRSP12, RNase-P/MRP	Hepatocellular carcinoma	^40–42^
Structure-mediated degradation	Cytoplasm	G3BP1, UPF1	\	^43^
TMAO-mediated degradation	Cytoplasm	TMAO	Dysbacteriosis in gut	^45^
Exosome-mediated degradation	Extracellular space	exosomes/ microvesicles	\	^48^
